# Field evaluation of natural human odours and the biogent-synthetic lure in trapping *Aedes aegypti*, vector of dengue and chikungunya viruses in Kenya

**DOI:** 10.1186/1756-3305-7-451

**Published:** 2014-09-23

**Authors:** Eunice A Owino, Rosemary Sang, Catherine L Sole, Christian Pirk, Charles Mbogo, Baldwyn Torto

**Affiliations:** International Centre of Insect Physiology and Ecology, P.O BOX 30772–00100, Nairobi, Kenya; Department of Zoology and Entomology, University of Pretoria, Pretoria, South Africa; Centre for Virus Research, Kenya Medical Research Institute, Nairobi, Kenya; Centre for Geographic Medicine Research – Coast, KEMRI & KEMRI – Wellcome Trust Research Programme, Kilifi, Kenya

**Keywords:** *Aedes aegypti*, Dengue, Chikungunya, Human odour, Mosquito, Traps

## Abstract

**Background:**

Methods currently used in sampling adult *Aedes aegypti*, the main vector of dengue and chikungunya viruses are limited for effective surveillance of the vector and accurate determination of the extent of virus transmission during outbreaks and inter - epidemic periods. Here, we document the use of natural human skin odours in baited traps to improve sampling of adult *Ae. aegypti* in two different endemic areas of chikungunya and dengue in Kenya – Kilifi and Busia Counties. The chemistry of the volatiles released from human odours and the Biogent (BG)-commercial lure were also compared.

**Methods:**

Cotton socks and T-shirts were used to obtain natural human skin volatiles from the feet and trunk of three volunteers (volunteers 1 and 2 in Kilifi and volunteers 2 and 3 in Busia). Using Latin square design, we compared the efficacies of BG sentinel traps baited with carbon dioxide plus (a) no bait, (b) human feet volatiles, (c) human trunk volatiles each against (c) a control (Biogent commercial lure) at the two sites. Coupled gas chromatography-mass spectrometry (GC-MS) was used to identify and compare candidate attractants released by the commercial lure and human odours.

**Results:**

*Ae. aegypti* captured in the trap baited with feet odours from volunteer 2 and trunk odours from the same volunteer were significantly higher than in the control trap in Busia and Kilifi respectively, [IRR = 5.63, 95% CI: 1.15 - 28.30, p = 0.030] and [IRR = 3.99, 95% CI: 0.95-16.69, p = 0.049]. At both sites, *Ae. aegypti* captures in traps baited with either the feet or trunk odours from volunteers 1 and 3 were not significantly different from the control. Major qualitative differences were observed between the chemical profiles of human odours and the commercial BG-lure. Aldehydes, fatty acids and ketones dominated human odour profiles, whereas the BG-lure released mainly hexanoic acid.

**Conclusions:**

Our results suggest that additional candidate attractants are present in human skin volatiles which can help to improve the efficacy of lures for trapping and surveillance of *Ae. aegypti*.

## Background

*Aedes aegypti* is one of the most important disease vectors worldwide. It is the principal vector of dengue [1], chikungunya [2] and Yellow fever [3] viruses. Among arboviral diseases, dengue fever has been reported to cause more human morbidity and mortality than any other arthropod-borne viral disease [4, 5]. It is estimated that each year, 50–100 million dengue infections and several hundred thousand cases of dengue hemorrhagic fever (DHF) occur, depending upon epidemic activity [6, 7]. In the past 10 years, there have been sporadic outbreaks of chikungunya fever along the Kenyan coast and the Indian Ocean islands of the Comoros, Seychelles, Reunion and Mauritius [8–11]. Additionally, in Kenya, a dengue outbreak was reported in Mandera County in September 2011 [12] and more recently in Mombasa County in May 2014 [13].

The increase in the emergence of dengue and chikungunya fever has been attributed to climate change [10] urbanization [4, 5, 14, 15] and globalization [4, 5, 16], amongst other factors. Consequently, the projected trends of continued global warming, urbanization and globalization will ensure that the incidence of these diseases will increase, especially if interventions are not forthcoming [15, 4]. Presently, there is no registered vaccine for prevention of dengue and chikungunya viruses which makes vector control the only available target for disease control and prevention. Under the circumstances, it is important to monitor the viruses and vector populations in endemic areas to understand their ecology before implementing appropriate and timely intervention. This therefore, calls for efficient surveillance and monitoring tools that will give reasonably accurate measures of disease and vector abundance data to guide decision on disease control measures.

The simplest and most effective sampling method for adult *Ae. aegypti* has been human-landing collections [17, 18]. Although effective in determining the exact anthropophilic species composition, human attack rate, and potential for disease transmission, renders this method inappropriate since it exposes the collectors to a degree of risk to infection and is also labour intensive. On the other hand, the popularly used mosquito surveillance trap, the Centers for Disease Control and Prevention (CDC) light trap [19] is virtually ineffective in sampling the day biting *Ae. aegypti* as it targets nocturnal host seeking species [17].

Odour-baited traps provide an effective means for monitoring insect populations. A recent study demonstrated the efficacy of an odour-baited trapping system for mosquito vectors of Rift Valley Fever virus [20, 21]. The Biogent (BG) sentinel trap baited with synthetic human skin compounds consisting of lactic acid, ammonia, and caproic acid (hexanoic acid) was used for sampling *Ae. aegypti* [22]. However, considerable reports have suggested that synthetic odours [23–25] or extracted human component blends [26] do not attract *Ae. aegypti* at a level comparable to natural human odours. Evaluating the effectiveness of the BG synthetic lure against natural human odours at different sites would therefore be critical for its wide scale use in disease vector control, especially *Aedes sp* vectors of chikungunya and dengue viruses.

In this study our objective was to compare the attractiveness of the commercial BG lure with natural human odours from two different sources, feet and trunk, in trapping *Ae. aegypti* in the field. We carried out this study in two dengue and chikungunya virus endemic areas in Kenya. Since mosquitoes are attracted to volatiles released from the different treatments, we compared the composition of these volatiles in order to identify the candidate attractants from the different treatments.

## Methods

### Study sites

**Figure 1:**
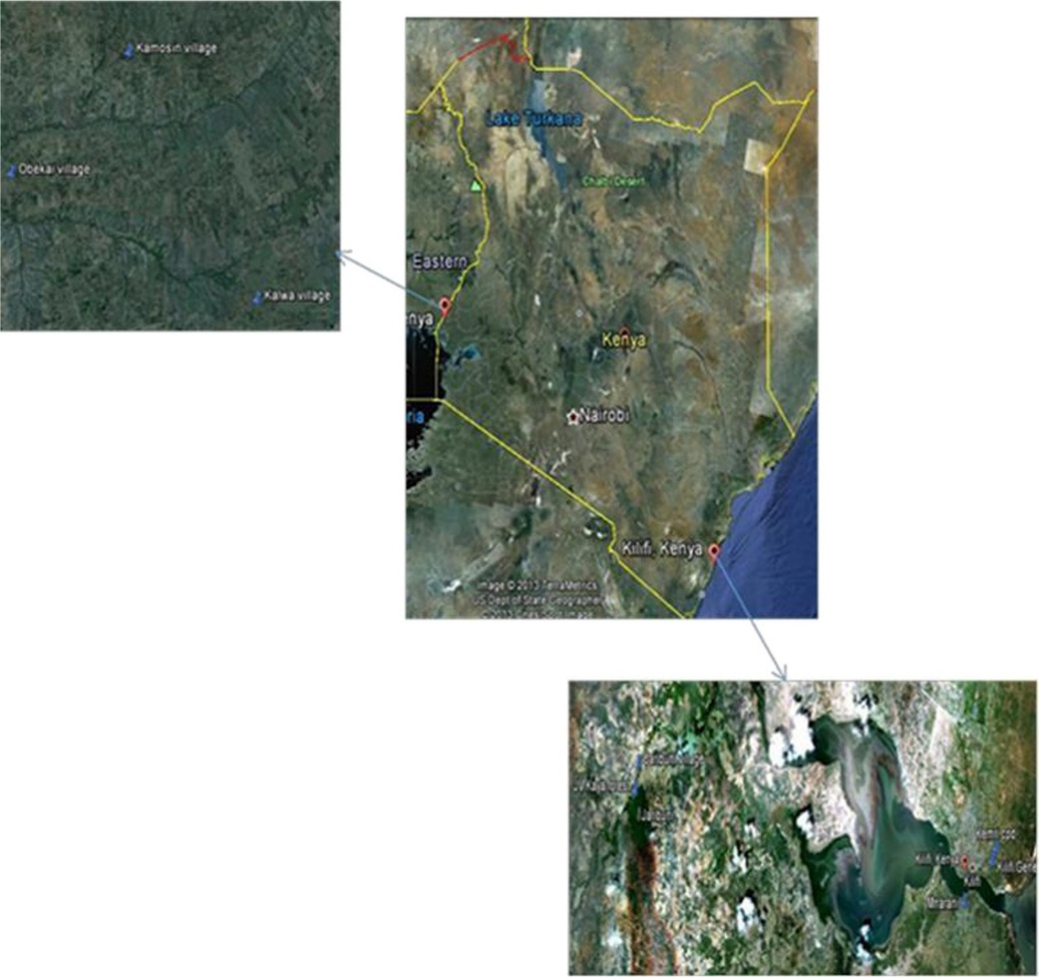
**The study sites; Kilifi district in the coast and Busia district in western Kenya.**

The study areas were Kilifi County at the Kenyan Coast and Busia County in Western Kenya (Figure 1). Previous seroprevalence studies had shown that dengue infection was prevalent in the Malindi area of Kilifi, with chikungunya infection occurring in Busia County [27]. The most recent outbreak of chikungunya also occurred at the coast [9].

Kilifi County has an average annual rainfall of 950 mm. The rainfall pattern is bimodal; the long monsoon rains (April - July) and the short rains (October- December). The annual temperatures range from a minimum of 21°C and a maximum of 32°C. Busia County on the other hand has an average annual rainfall of 1500 mm. The rainfall pattern is also bimodal; long rains (March - June) and short rains between (October -December). The temperatures range from a minimum of 14°C and a maximum of 30°C.

In Busia County, traps were set up in villages in the rural areas namely Obekai (30.875 N, 34 12.293 E), Kamosin (0 31.530 N, 34 13.125E) and Kalwa (0 30.190 N, 3414.020E). These are locations that occur at approximately 1189 m above the sea level (ASL). The main vegetation in these areas consists of large, tall eucalyptus trees that form thick canopies. The local inhabitants are mainly small-scale farmers growing maize, millet and cassava food crops while a few grow sugarcane and coffee as cash crops. They also keep a few animals mainly cattle, sheep, goats, pigs, chicken and guinea fowls.

In Kilifi county, traps were set up at two sites located in the urban area; Kenya medical research institute (KEMRI) campus in Kilifi (3 37.800 S, 39 51.483 E) and Mnarani estate (3 38.368 S, 39 50.824 E), while the other site was in the Kaya Kauma forest (3 37.183 S, 39 44.167 E). These are locations that occur at approximately 30.5 m ASL. The inhabitants in the urban area mainly engage in small businesses or work in offices. They also grow maize, cassava and sweet potatoes and keep a few animals, mainly goats.

The traps were set up during the wet seasons at both sites. In Kilifi, the traps were set up in April 2012 and June 2012 while in Busia they were set up in December 2012 and April 2013.

### Study design

A Latin square design was used. At each sampling location, Kilifi or Busia, the efficacy of the BG sentinel trap baited with carbon dioxide plus (i) the BG commercial lure, (ii) cotton socks or T-shirts worn by two volunteers in Kilifi and two volunteers in Busia and (iii) no bait, were set.

### Odour collection and mosquito sampling with odour-baited traps

**Figure 2:**
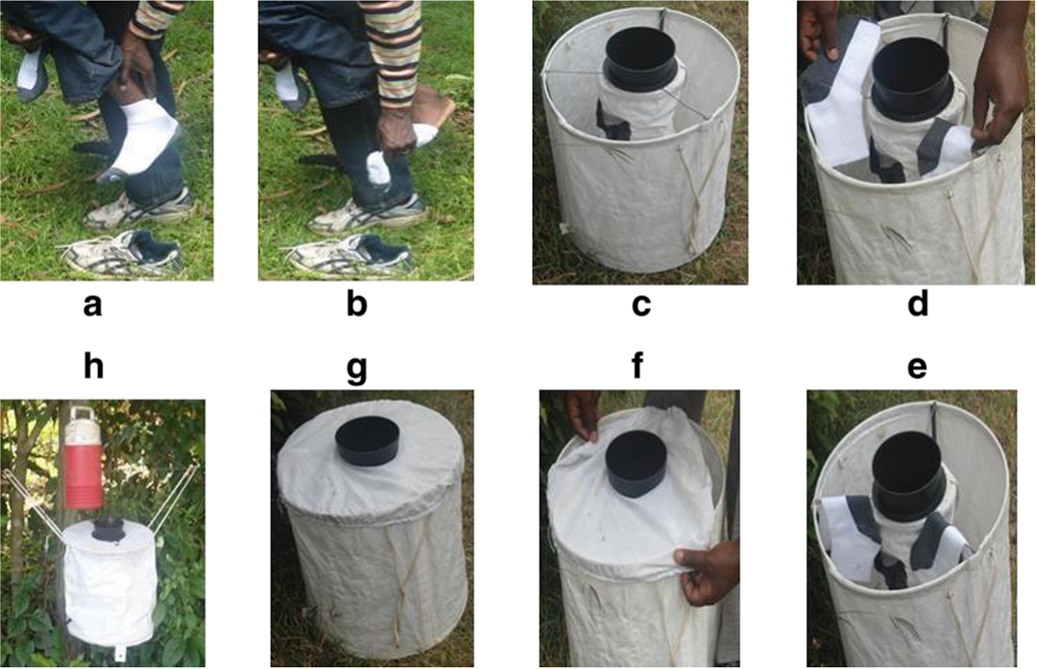
**The BG sentinel traps were baited with socks and set up in Busia and Kilifi counties of Kenya.** Assembly follows steps **a-h**.

Odours were obtained from the feet and trunk of three male volunteers (volunteers 1, 2 and 3) aged between 25–50 years. Trunk and feet odours from volunteer 1 were used as baits in Kilifi and that from volunteer 3 in Busia while that from volunteer 2 were used in both Kilifi and Busia. The same individuals were involved throughout the study. Socks and T-shirts worn by volunteers 1 and 2 were used to sample mosquitoes in Kilifi while those worn by volunteers 2 and 3 were used to sample mosquitoes in Busia. The volunteers were requested to put on new, clean, 100% cotton socks and T-shirts (Lux Industries Ltd 39 K.K Tagare st, Kolkata-700-007) for 18 hrs daily to trap odours from their feet and trunk for nine 12 days. After 18 hrs each day, the volunteers removed the socks and T-shirts which were used to bait BG sentinel traps by hanging them on the rails of the BG sentinel trap inner structure as shown in (Figure 2). During this period and prior to wearing the socks and T-shirts, the volunteers were provided with an odourless soap daily for bathing and were requested to avoid the use of deodorants and/or perfumes.

### Mosquito sampling with odour baited traps

Four different sites were randomly chosen around homesteads after obtaining oral consents from the homestead heads. Four BG sentinel traps baited with the commercial lure, socks or T-shirts worn by the different volunteers and no baits were randomly set up at each of the four sites with a distance of at least one hundred (100 m) between traps. The traps were hung at 0.2 m above the ground and attached to each was a Bioquip igloo that dispensed carbon dioxide in the form of dry ice (Figure 2). To account for positional effects, traps were rotated every experimental day. This was repeated for 12 days.

Because some sites were at a distance of up to 40 km apart in both Busia and Kilifi, traps were set up at each site at different times of the day and left to run for 24 hrs. Mosquitoes were then collected and transported to the laboratory where they were freeze-killed and identified under a dissecting microscope to species level using morphological keys [28–31]. Mosquitoes were categorized as engorged when blood fed or gravid based on observation of their abdominal condition as described in the WHO Manual [32]. Daily mosquito counts per trap were recorded for each mosquito species.

### Collection and analysis of volatiles

In order to analyze and compare the composition of volatiles released by the commercial lure and the human odours, headspace volatiles from the commercial BG-lure and from the three volunteers’ feet and trunks were collected using solid phase micro- extraction (SPME) technique for 6 hrs at room temperature. Odours were also trapped and analyzed from unused 100% cotton socks and T-shirts, which acted as control. The odours were adsorbed on 75 μm carboxen-poly dimethyl siloxane (CAR/PDMS) and 50/30 μm Divinyl benzene/ Carboxen/ Poly dimethyl siloxane (DVB/CAR/PDMS) (Supelco: Sigma-Aldrich Pty Ltd, Bellefonte, USA) fibers. The fibers were each conditioned at 270°C for 1 hr before use.

After extraction the SPME fibers were injected into the gas chromatography - mass spectrometry (GC-MS) and mass selective detector (MSD) system consisting of a model HP 7890A gas chromatograph, a 5975 Mass spectrometer with a triple Axis detector and an Agilent ChemStation data system. The GC column was a Carbowax HP-20 with 20% Carbowax stationary phase (30 m × 250 μm × 0.25 μm film thickness). The carrier gas was helium with a column head pressure of 8.8271 psi and flow rate of 1.2 ml/min. Inlet temperature was 220°C and mass selective detector temperature was 230°C. The oven temperature was held at 35°C for 5 min, a rise of 10°C min^−1^ to a final temperature of 220°C, which was held for 20.5 min. The identity of compounds in the volatiles was determined by comparison with references from mass spectral libraries (NIST05, Agilent Technologies [NIST database, G1033A, revision D.05.01, ChemStation data system (G1701EA, version E.02.00) and SPME analysis of a mixture of the authentic compounds. Each compound in the authentic mixture was 100 ng/ul.

The chemicals were ; hexanoic acid, hexanal, octanal, nonanal, decanal, 6-methyl-5-hepten-2-one, geranylacetone (Sigma-Aldrich Chemie GmbH, Germany), 3-methylbutyric acid and 2-methylpropionic acid (Sigma-Aldrich Corporation, 3050 Spruce Street, St. Louis, Missouri 63103 USA). Purities of the compounds ranged between 95% and 99%.

### Data analysis

The daily mosquito counts in the different traps were subjected to negative binomial regression following the generalized linear models (GLM) procedures in R 3.1.0 [33]. The BG commercial lure baited trap was used as the reference category. The incidence rate ratios (IRR) - a likelihood measure that mosquito species chose other treatments instead of the control - and corresponding P-values were estimated. The chi-square test was applied to evaluate differences between proportions of male and female *Ae. aegypti* per trap and differences between proportions of fed and gravid mosquitoes per a treatment trap and the control. The tests were performed at 5% significance level.

### Ethics statement

The study was approved by the national ethics review committee based at the Kenya Medical Research Institute (KEMRI) and informed consent was obtained from each of the participants.

## Results

### Mosquito sampling with odour baited traps

A total of 1,989 *Ae. aegypti* were collected, 1,805 in Kilifi and 184 in Busia. Overall, we found a significant variation in trap captures of *Ae. aegypti* based on location [X^2^ = 332.35, d.f = 1, p < 0.001], with higher trap captures recorded in Kilifi than Busia for the same number of days [IRR = 9.81, 95% CI: 5.8-16.6, p < 0.001].

**Figure 3:**
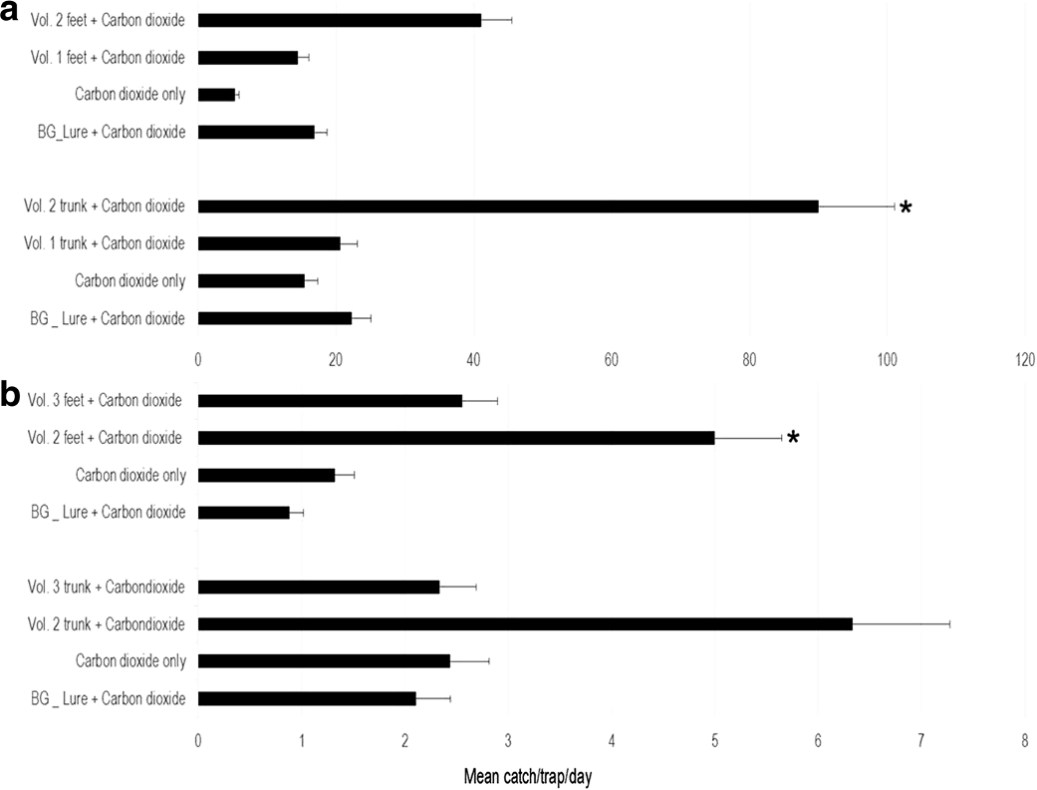
**The mean number/day and ± S.E of** ***Aedes aegypti*** **captured by the various BG sentinel traps baited with different baits in Kilifi and Busia counties.** The different panels show comparisons at the two locations; Panel **a** –Kilifi and Panel **b**- Busia. Asterisks indicate that the mean catch of the trap is significantly different from the mean catch of the control trap (Biogent’s commercial lure baited trap). Error bars indicate standard error of the mean.

In Kilifi, the trap baited with trunk volatiles from volunteer 2 trapped a significantly higher number of *Ae. aegypti* than the control (Figure 3), [IRR = 3.99, 95% CI: 0.95-16.69, p = 0.049], while the trap baited with trunk volatiles from volunteer 1 and the trap baited with carbon dioxide only captured fewer of this mosquito species than the control [IRR = 0.92, 95% CI: 0.22 - 3.87] and [IRR = 0.691, CI: 0.16 - 2.92] respectively (Table 1). At the same site, the trap baited with feet volatiles from volunteer 2 captured more *Ae. aegypti* than the control trap (Figure 3), [IRR = 2.43, 95% CI: 0.71 - 8.29], while both the trap baited with feet volatiles from volunteer 1 and the trap baited with carbon dioxide only captured fewer *Ae. aegypti* than the control trap [IRR =0.86, 95% CI: 0.25 - 2.93] and [IRR = 0.32, 95% CI: 0.09 -1.10] respectively (Table 1).

**Table 1:** **Comparisons of mosquito collections by BG sentinel traps baited with feet and trunk odours from volunteer 1, volunteer 2 and carbon dioxide in Kilifi county and from volunteer 2, volunteer 3 and carbon dioxide in Busia county relative to the control (Biogents commercial lure baited BG sentinel trap) trap**

Site	Treatment	IRR(95%CI)	P value	Treatment	IRR(95%CI)	P value
Kilifi	Carbon dioxide	0.69(0.16-2.92)	0.602	Carbon dioxide	0.32(0.09-1.10)	0.064
Kilifi	Volunteer 1 trunk odour	0.92(0.22-3.87)	0.906	Volunteer 1 feet odour	0.86(0.25-2.93)	0.790
Kilifi	Volunteer 2 trunk odour	3.99(0.95-16.69)	0.049*	Volunteer 2 feet odour	2.43(0.71-8.29)	0.143
Busia	Carbon dioxide	1.16(0.19 - 6.97)	0.867	Carbon dioxide	1.50(0.28-8.04)	0.627
Busia	Volunteer 2 trunk odour	3.00(0.52-17.61)	0.203	Volunteer 2 feet odour	5.63(1.15-28.30)	0.030*
Busia	Volunteer 3 trunk odour	1.10(0.18 -6.67)	0.909	Volunteer 3 feet odour	2.87(0.57-14.80)	0.192

Estimated incidence rate ratio (IRR); confidence interval (CI) and corresponding P-values based on comparison to the BG lure following generalized linear model (GLM) with negative binomial error structure and log link in R 3.1.0 software. The IRR for the control is 1; values above this indicate better performance while values below indicate under performance relative to the control. Asterisks on p values indicate significant difference with the control.

In Busia, the traps baited with foot odour from volunteer 2, foot odour from volunteer 3 and carbon dioxide only captured more *Ae. aegypti* than the control (Table 1), with the trap baited with feet volatiles from volunteer 2 trapping significantly more *Ae. aegypti* than the control trap (Figure 3) [IRR = 5.63, 95% CI: 1.15 - 28.30, p = 0.030]. The same trend was observed when traps baited with the same volunteer’s trunk volatiles were compared with the control (Figure 3). The order of performance was; volunteer 2 [IRR = 3.00, 95% CI: 0.18 - 6.68], carbon dioxide only [IRR =1.16, 95% CI: 0.192- 6.98] and volunteer 3 [IRR = 1.11, 95% CI: 0.51-17.61] (Table 1)].

When proportions of male and female *Ae. aegypti* captured per trap were compared, a significantly higher number of males were captured by the trap baited with trunk odours from volunteer 1 [p < 0.001, X^2^ = 20.92, d.f = 1] (Table 2). A further comparison between the proportions of fed and gravid *Ae. aegypti* per treatment trap and the control trap showed that traps baited with foot odours from volunteer 1, volunteer 2 and volunteer 3 captured more gravid *Ae. aegypti* than the control (Table 2).

**Table 2:** **Comparisons of** ***Ae aegypti*** **proportions per trap by sex and abdominal status with corresponding catch indices (CI)**

Bait	Total	♂ Proportion	♀ Proportion	P-values	Fed proportion	CI	P-values	Gravid proportion	CI	P- values
BG-Lure	191	52.4^a^	47.6^a^	0.412	5.5	1	-	2.2	1	-
Carbon dioxide only	166	45.8^a^	54.2^a^	0.153	2	0.3	0.191	3.3	1.3	0.951
Volunteer 2 socks	415	41.9^b^	58.1^a^	<0.001	0.8	0.2	0.040*	1.2	1.3	1
Volunteer 2 T-shirt	858	52.0^a^	48.0^a^	0.112	0.2	0.2	0.001*	1.2	2.5	1
Volunteer 1 socks	130	43.9^a^	56.1^a^	0.061	0	0	<0.001*	2.7	1	1
Volunteer 1 T-shirt	185	62.2^b^	37.8^a^	<0.001	0	0	<0.001*	0	0	0.252
Volunteer 3 socks	23	43.5^a^	56.5^a^	0.562	0	0	<0.001*	7.7	4.5	0.043*
Volunteer 3 T-shirt	21	0^b^	100^a^	<0.001	0	0	<0.001*	0	0	0.256

Proportions following each other in the rows with different letters (a and b) are significantly different from each other. Asterisks on p values indicate significant difference with the control. The P-values are based on pair-wise comparison following chi-square goodness-of-fit in R 3.1.0 software.♂-Male *Ae. aegypti*, ♀- female *Ae. aegypti*.

Although data analysis was only limited to *Ae. aegypti,* other mosquito species including *Culex quinquefasciatus*, *Culex annulioris, Anopheles gambiae* and *Anopheles funestus* also occurred in large numbers in the traps at both sites. There were also small numbers of *Anopheles coustanii* in both Kilifi and Busia, *Mansonia uniformis, Mansonia africana*, *Eretmapodites chrysogaster group*, *Culex poicilipes, Coquillettidia faseri*, *Aedes metallicus*, *Aedes woodi* and *Aedes bromeliae* in Kilifi.

### Analysis of volatiles

The BG-lure, trunk and feet of human volunteers all released volatiles that attracted *Ae. aegypti* into traps. Analysis of the volatiles showed major qualitative and quantitative differences in the chemical profiles between trunk and foot odours and the commercial lure. Aldehydes and fatty acids dominated the volatiles released by human odours, which varied between individual volunteers, whereas hexanoic acid was the major component released by the BG lure (Table 3).

**Table 3:** **Main compounds identified in the volatiles released by the commercial BG-lure and trunk and feet of human volunteers captured on SPME and analyzed coupled GC-MS analysis**

Volatile source	Major compounds in percentages
BG-Lure	Hexanoic acid 73%
Volunteer 1, 2 & 3 trunks	Decanal (8% -33%)
	Hexanal (8 - 32%)
	6-methyl-5-hepten-2-one(15 - 28%)
	Nonanal (2 - 26%)
	Geranylacetone (3 - 13%)
	Hexanoic acid (4 - 9%)
Volunteer 1, 2 & 3 feet	Hexanoic acid (7-36%)
	Octanal (3 – 18%)
	Nonanal (7 - 17%),
	Hexanal (3 -15%)
	3-methylbutyric acid (7 - 9%)
	2-methylpropionic acid (2-9%)

## Discussion

We observed that *Ae. aegypti* captures in Kilifi were generally higher than in Busia. Several factors could have played a role in this difference. Firstly, *Ae. aegypti* is a known container breeding mosquito [34, 35] and since the sampling sites in Kilifi were mainly in an urban area, there is the likelihood for the mosquito to find more of this type of breeding site in this area. On the other hand, the Busia sampling sites which are rural would provide the opposite situation. Secondly, Kilifi being an old urban center, with older and abundant houses that could serve as suitable breeding sites for this mosquito species. Walker *et al*., 2008 [36] observed that older houses with mature vegetation, and objects collected in the yard tended to have higher densities of *Ae. aegypti* eggs than newer houses. Thirdly, previous studies of *Ae. aegypti* in the Kenyan coast observed that they are highly anthropophillic and domesticated in behavior, where their life cycle transpires mainly inside and around human residences [37, 38]. They are therefore more likely to be attracted to human odours than the inland populations of Busia. Furthermore, *Ae. aegypti* mosquitoes have been observed to be highly adapted to urban rather than rural areas. They have a preference to rest inside houses and for areas with high human density, a behaviour that favours vector-human contact [39, 40]. Therefore, as an urban area, a higher population density in Kilifi could have contributed to a higher abundance of *Ae. aegypti.*

Climatic differences between the two sites could also have contributed to the observed variation. Busia receives an average annual rainfall of 1500 mm and is cooler with a minimum temperature of 14°C and a maximum of 30°C compared to an average annual rainfall of 950 mm and higher temperatures with a minimum of 21°C and a maximum of 32°C in Kilifi. Previous studies reported that while adequate amounts of rain will create natural water bodies and fill artificial habitats, providing females with opportunities to lay their eggs, excessive rain may flush the immature stages, especially the eggs, from their habitats causing a population crash of *Ae. aegypti* [41]. It has also been observed that higher temperatures increased the developmental rate of *Ae. aegypti* [42], thus Kilifi which is relatively warmer than Busia would favour the breeding of higher densities of *Ae. aegypti* than Busia.

The traps baited with natural human odours from the feet and trunk, especially from volunteer 2 captured significantly more *Ae. aegypti* than the control trap baited with the synthetic commercial lure. Similar results were observed when the efficacy of the BG-sentinel trap baited with the commercial lure was compared with human landing/biting collections, a gas-powered CO_2_ trap, and a Fay-Prince trap, in monitoring adult populations of *Ae. aegypti* in field tests in the city of Belo Horizonte, Brazil [43]. Furthermore, human odours were found to be significantly more attractive than a synthetic three-component blend consisting of L-Lactic acid, acetone and dimethyl disulfide during competitive bioassays that simultaneously compared the attractiveness of *Ae. aegypti* to two treatments in a dual port olfactometer [26]. The presence of additional fatty acids such as 2-methylpropionic acid and 3-methylbutyric acid, the four aldehydes; hexanal, octanal, nonanal and decanal and the two ketones 6-methyl-5-hepten-2-one and geranylacetone in human odours but not the BG-lure, suggests that these compounds likely played a role in the attractiveness of human odours over the BG-lure. Indeed, previous studies had shown that some of these compounds, including 2-methylpropionic acid, 3-methylbutyric acid, hexanal, octanal, nonanal and decanal are attractants of other mosquito species such as the malaria mosquito *Anopheles gambiae* [44, 45] and mosquito vectors of Rift Valley Fever virus [21].

We found individual variation in the attractiveness of volunteers to mosquitoes based on our field captures. This observation is supported by our chemical analysis of volatiles collected from the different individuals, which showed qualitative and quantitative differences in specific components. This result is similar to previous studies of volatiles of mammalian odours in mosquito attraction [46, 47]. For example, the difference in the attraction of different individuals to host seeking *Ae. aegypti* has been attributed to the difference in the quantity of lactic acid present on their skin [48]. Individuals with higher amounts of lactic acid on their skin attracted more mosquitoes, while adding lactic acid to the skin rubbings of individuals who were less attractive made them more attractive to mosquitoes. Inter-individual variation in body odour has also recently been attributed to the aggregation of different communities of micro biota on the skin. It has been demonstrated that individuals with lower bacteria diversity and with a significantly higher abundance *of Leptotrichia spp*., *Delftia spp.* and *Actinobacteria Gp3 spp* of bacteria on their skin are highly attractive to *Anopheles gambiae s.s*. while individuals with a higher microbial diversity and a higher abundance of *Pseudomonas spp* or *Variovorax spp.* of bacteria on their skin are poorly attractive [49].

The fact that traps baited with natural human skin odours collected significantly more male *Ae. aegypti* than the trap baited with the Biogent’s lure is striking. This suggests that having a trap that is efficient in capturing male *Ae. aegypti* would help dengue and chikungunya fever control programs because it has been established that although male *Ae. aegypti* are not blood feeders they are usually infected with dengue and chikungunya viruses via transovarial transmission [50, 51]. Recent studies document that male mosquitoes play an important role in the prevalence and maintenance of these diseases in the environment through venereal transmission of chikungunya virus from male to female *Ae. Aegypti,* which then transmits it to possible vertebrate hosts [52].

Lastly, the observation that traps baited with volatiles from the feet of volunteers not only captured more gravid *Ae. aegypti* than the control trap but also some blood fed ones increases their potential usefulness in dengue and chikungunya fever surveillance. Gravid mosquitoes are a high priority in arboviral surveillance programs. Conceivably, gravid mosquitoes would have already been exposed to virus infection through previous feeding, hence serving as likely indicators of virus activity [53]. On the other hand, blood-fed mosquitoes give information regarding the feeding preference, seroconversion status of that host, and infectivity level of the reservoir host, [54], which immensely helps researchers in understanding the ecology of arboviruses spread by mosquitoes. Additionally, testing of blood fed mosquitoes helps to understand the interaction mechanisms between host, vector and possible reservoirs, and to identify and evaluate the role of potential bridge vector species in transmission of pathogens of public health importance [55].

## Conclusions

Our data indicate that traps baited with natural skin volatiles are more efficient than traps baited with the Biogent synthetic lure in sampling *Ae. aegypti*. However, the efficacy of human odours varies between individuals [47, 56, 57] and hence causes variation in trap captures. Additional studies will be required to determine the specific compound(s) that increase the attractiveness of human odours and subsequent trap captures for development and evaluation.

## References

[1] Gubler DJ, Monath TP (1989). Dengue. The Arboviruses: Epidemiology and Ecology, vol 2.

[2] Halstead SB, Nimmannitya S, Yamarat C, Russell PK (1967). Hemorrhagic fever in Thailand; recent knowledge regarding etiology. Jpn J Med Sci Biol.

[3] Monath TP, Monath TP (1989). Yellow fever. The Arboviruses: Epidemiology and Ecology, vol 5.

[4] Gubler DJ (2002). The global emergence/resurgence of arboviral diseases as public health problems. Arc Med Res.

[5] Gubler DJ, Gubler DJ, Kuno G (1997). Dengue and dengue hemorrhagic fever: its history and resurgence as a global public health problem. Dengue and Dengue Hemorrhagic Fever.

[6] World Health Organization (2006). Report of the scientific working group on dengue. Special Programme for Research and Training in Tropical Diseases.

[7] World Health Organization (2009). Guidelines for diagnosis, treatment, prevention and control. Dengue.

[8] Powers AM, Logue CH (2007). Changing patterns of Chikungunya virus: reemergence of a zoonotic arbovirus. J Gen Virol.

[9] Sergon K, Njuguna C, Kalani R, Ofula V, Onyango C, Konongoi LS, Bedno S, Burke H, Dumilla AM, Konde J (2008). Seroprevalence of Chikungunya virus (CHIKV) infection on Lamu Island, Kenya, October 2004. Am J Trop Med Hyg.

[10] Chretien JP, Anyamba A, Bedno SA, Breiman RF, Sang R, Sergon K, Powers AM, Onyango CO, Small J, Tucker CJ, Linthicum KJ (2007). Drought associated Chikungunya emergence along coastal East Africa. Am J Trop Med Hyg.

[11] Sang RC, Ahmed O, Faye O, Kelly CL, Yahaya AA, Mmadi I, Toilibou A, Sergon K, Brown J, Agata N, Yakouide A, Ball MD, Breiman RF, Miller BR, Powers AM (2008). Entomologic investigations of a Chikungunya virus epidemic in the union of the Comoros 2005. Am J Trop Med Hyg.

[12] **State-confirms-outbreak-of-dengue-fever-in-mandera** [ http://www.standardmedia.co.ke/?articleID=2000043805&story_title=state-confirms-outbreak-of-dengue-fever-in-mandera]

[13] **Coast region hit by mosquito transmitted fever****[**http://www.standardmedia.co.ke/mobile/?articleID=2000121930&story_title=coast-region-hit-by-mosquito-transmitted-fever]

[14] Beatty ME, Letson GW, Margolis HS (2008). Estimating the global burden of dengue. Abstract Book: Dengue. The Second International Conference on Dengue and Dengue Haemorrhagic Fever.

[15] Alirol E, Getaz L, Stoll B, Chappuis F, Loutan L (2009). Urbanization and infectious disease in a globalized world. Lancet Infect Dis.

[16] Enserink M (2007). Infectious diseases: Chik*un*gunya: no longer a third world disease. Sci.

[17] Service M W (1993). Mosquito Ecology: Field-sampling Methods.

[18] Focks DA (2003). A review of entomological sampling methods and indicators for dengue vectors. Special Programme for Research and Training in Tropical Diseases.

[19] Sudia WR, Chamberlain W (1962). Battery operated light trap, an improved model. Mosq News.

[20] Tchouassi DP, Sang R, Sole CL, Bastos ADS, Mithoefer K, Torto B (2012). Sheep skin odor improves trap captures of mosquito vectors of rift valley fever. PLoS Negl Trop Dis.

[21] Tchouassi DP, Sang R, Sole CL, Bastos ADS, Teal PEA, Borgemeister C, Torto B (2003). Common host-derived chemicals increase catches of disease-transmitting mosquitoes and can improve early warning systems for rift valley fever virus. PLoS Negl Trop.

[22] Geier M, Rose A, Grunewald J, Jones O (2006). New mosquito traps improve the monitoring of disease vectors. Int Pest Control.

[23] Canyon DV, Hii JLK (1997). Efficacy of carbon dioxide, 1-octen-3-ol, and lactic acid in modified Fay-Prince traps as compared to man-landing catch of *Aedes aegypti*. J Am Mosq Control Assoc.

[24] Jones JW, Sithiprasasna R, Schleich S, Coleman RE (2003). Evaluation of selected traps as tools for conducting surveillance for adult *Aedes aegypti* in Thailand. J Am Mosq Control Assoc.

[25] Schoeler GB, Schleich SS, Manweiler SA, Sifuentes VL (2004). Evaluation of surveillance devices for monitoring *Aedes aegypti* in an urban area of northeastern Peru. J Am Mosq Control Assoc.

[26] Bernier UR, Kline DL, Allan SA, Barnard DR (2007). Laboratory comparison of *Aedes aegypti* attraction to human odors and to synthetic human odor compounds and blends. J Am Mosq Control Assoc.

[27] Mease LE, Coldren RL, Musila LA, Prosser T, Ogolla F, Ofula VO, Schoepp RJ, Rossi CA, Adungo N (2011). Seroprevalence and distribution of arboviral infections among rural Kenyan adults: a cross-sectional study. Virol J.

[28] Edwards FW (1941). Mosquitoes of the Ethiopian Region III.

[29] Gillies MT, DeMeillon B (1968). The Anophelinae of Africa South of the Sahara (Ethiopian Zoogeographical Region).

[30] Huang YM, Ward RA (1981). A pictorial key for the identification of the mosquitoes associated with yellow fever in Africa. Mosq Systematic.

[31] Rueda LM (2004). Pictorial keys for the identification of mosquitoes (Diptera; Culicidae) associated with dengue virus transmission. Zoo Taxa.

[32] World Health Organization (2003). Malaria entomology and vector control. Learner’s Guide.

[33] Core Team R: *A Language and Environment for Statistical Computing. R Foundation for Statistical Computing*. Vienna, Austria; URL http://www.R-project.org/ 2014

[34] Christophers SR (1960). Aedes aegypti (L.), the Yellow Fever Mosquito: its Life History, Bionomics and Structure.

[35] Southwood TRE, Murdie G, Yasuno M, Tonn RJ, Reader PM (1972). Studies on the life budget of *Aedes aegypti* in Wat Samphaya, Bangkok, Thailand. Bull World Health Organ.

[36] Walker KR, Joy TK, Ellers-KIrk C, Ramberg FB (2011). Human and environmental factors affecting *Aedes aegypti distribution* in an arid urban environment. J Am Mosq Control Assoc.

[37] Tabachnick WJ, Powell JR (1978). Genetic structure of the East African domestic populations of *Aedes aegypti*. Nature.

[38] McDonald PT (1977). Population characteristics of domestic *Aedes aegypti* (diptera: culicidae) in villages on the Kenya coast. II. Dispersal within and between villages. J Med Entomol.

[39] Lima-Camara TN, Honório NA, Lourenço-de-Oliveira R (2006). Frequency and spatial distribution of *Aedes aegypti* and *Aedes albopictus* (Diptera, Culicidae) in Rio de Janeiro, Brazil. J Pub Health.

[40] Tsuda Y, Suwonkerd W, Chawprom S, Prajakwong S, Takagi M (2006). Different spatial distribution of *Aedes aegypti* and *Aedes albopictus* along an urban rural gradient and the relating environmental factors examined in three villages in northern Thailand. J Am Mosq Control Assoc.

[41] Koenraadt CJ, Harrington LC (2008). Flushing effect of rain on container-inhabiting mosquitoes *Aedes aegypti* and *Culex pipiens* (Diptera: Culicidae). J Med Entomol.

[42] Yusoff N, Budin H, Ismail S (2012). Simulation of population dynamics of *Aedes aegypti* using climate dependent model. A W Acad Sci, Eng Tech.

[43] Kröckel U, Rose A, Eiras AE, Geier M (2006). New tools for surveillance of adult yellow fever mosquitoes: comparison of trap catches with human landing rates in an urban environment. J Am Mosq Control Assoc.

[44] Mukabana WR, Mweresa CK, Otieno B, Omusula P, Smallegange RC, Van Loon JAJ, Takken W (2012). A novel synthetic odorant blend for trapping of malaria and other African mosquito species. J Chem Ecol.

[45] Nyasembe VO, Teal PEA, Mukabana WR, Tumlinson J, Torto B (2012). Behavioural response of the malaria vector *Anopheles gambiae* to host plant volatiles and synthetic blends. Parasit Vectors.

[46] Lindsay SW, Adiamah JH, Miller J (1993). Variation in attractiveness of human subjects to malaria mosquitoe*s (Diptera: Culic*idae) in the Gambia. J Med Entomol.

[47] Knols BGJ, De Jong R, Takken W (1995). Differential attractiveness of isolated humans to mosquitoes in Tanzania. Trans R Soc Trop Med Hyg.

[48] Geier M, Sass H, Boeckh J (1996). A Search for Components in Human Body Odors that Attract Females of Aedes aegypti. Ciba Foundation Symposium 200.

[49] Verhulst NO, Qiu YT, Beijleveld H, Maliepaard CA, Knights D, Schulz S, Berg-Lyons D, Lauber CL, Verduijn W, Haasnoot GW, Mumm R, Bouwmeester HJ, Claas FHJ, Dicke M, JJA V l, Takken W, Knight R, Smallegange RC (2011). Composition of human skin micro biota affects attractiveness to malaria mosquitoes. PLoS One.

[50] Thenmozhi V, Tewari SC, Manavalan R, Balasubramanian A, Gajanana A (2000). Natural vertical transmission of dengue viruses in *Aedes aegypti* in southern India. Trans R Soc Trop Med Hyg.

[51] Thavara U, Tawatsin A, Pengsakul T, Bhakdeenuan P, Chanama S, Anantapreecha S, Molito C, Chompoorsri J, Thammapalo S, Sawaboabyakert OM, Siriyasatien P (2009). Outbreak of chikungunya fever in Thailand and virus detection in field population of vector mosquito *Aedes aegypti* and *Aedes albopictus*. J Trop Med Public Health.

[52] Mavale M, Parashar D, Sudeep A, Gokhale M, Ghodke Y, Geevarghese G, Arankalle V, Chandra AM (2010). Venereal transmission of chikungunya virus by *Aedes aegypti* mosquitoes. Am J Trop Med Hyg.

[53] Allan BF, Goessling LS, Storch GA, Thach RE (2010). Blood meal analysis to identify reservoir hosts for *Amblyomma americanum* ticks. Emerg Infect Dis.

[54] Kay BH, Boyd AM, Ryan PA, Hall RA (2007). Mosquito feeding patterns and natural infection of vertebrates with Ross river and Barmah forest viruses in Brisbane, Australia. Am J Trop Med Hyg.

[55] Reiter P (1983). A portable battery-powered trap for collecting gravid *Culex* mosquitoes. Mosq News.

[56] Logan JG, Birkett AM, Clark SJ, Powers S, Seal NJ, Wadhams LJ, Mordue AJ, Pickett JA (2008). Identification of human-derived volatile chemicals that interfere with attraction of *Aedes aegypti* mosquitoes. J Chem Ecol.

[57] Schreck CE, Kline DL, Carlson DA (1990). Mosquito attraction to substances from the skin of different humans. J Am Mosq Control Assoc.

